# Nonvitamin K Anticoagulants: Risk of Bleeding When Interacting With Other Medications: A Cohort Study From Medicare

**DOI:** 10.1002/clc.70023

**Published:** 2024-10-03

**Authors:** Ainhoa Gomez‐Lumbreras, Madeline Brendle, Krystal Moorman‐Bishir, Malinda Tan, Daniel C. Malone

**Affiliations:** ^1^ Department of Pharmacotherapy University of Utah Salt Lake City Utah USA; ^2^ Department of Health Outcomes, College of Pharmacy The University of Texas at Austin Austin Texas USA; ^3^ Real World Evidence, Open Health Bethesda Maryland USA

**Keywords:** drug interactions, factor Xa inhibitors, gastrointestinal hemorrhage, hemorrhage, risk factors

## Abstract

**Introduction:**

Patients on nonvitamin K antagonist (NVKA) are usually taking other drugs. Potential interaction may increase the gastrointestinal (GI) bleeding risk associated with NVKA.

**Methods:**

Observational cohort study using Medicare data from 2017 to 2020. Participants receiving a NVKA were included. A concomitant overlapping period while on NVKA was assessed for nonsteroidal anti‐inflammatory drugs (NSAIDS), selective serotonin reuptake inhibitors (SSRI), antiplatelets, glucocorticoids, aspirin and proton pump inhibitors (PPI). A logistic regression predicting either any bleeding or GI bleeding was conducted estimating the odds ratio (OR) and 95% confidence interval (CI).

**Results:**

A total of 102 531 people on NVKA with mean age 77 years (SD = 9.8) and 55% females (*N* = 56 671) were included. Previous history of GI bleeding occurred in 2 908 (2.8%) participants, concomitant exposure to PPI occurred in 38 713 (38%), SSRI in 16 487 (16%), clopidogrel in 15 795 (15.4%), NSAIDs in 13 715 (13.4%) and glucocorticoids in 13 715 (13.4%). Risk for any bleeding was shown for clopidogrel (OR: 1.37, 95% CI: 1.30, 1.44), prasugrel/ticagrelor (OR: 1.36, 95% CI: 1.18, 1.58), glucocorticoids (OR: 1.26, 95% CI: 1.19, 1.34), and SSRIs (OR: 1.13, 95% CI: 1.07, 1.19). GI bleeding risk was shown for clopidogrel (OR: 1.44, 95% CI: 1.34, 1.55), prasugrel/ticagrelor (OR: 1.47, 95% CI: 1.20, 1.79), SSRIs (OR: 1.09, 95% CI: 1.01, 1.17) and glucocorticoids (OR: 1.33, 95% CI: 1.23, 1.44). PPI use was correlated with both NSAID (*r* = 0.07, *p* ≤ 0.0001) and SSRI use (*r* = 0.09, *p* ≤ 0.0001).

**Conclusion:**

NVKA concomitantly taken with antiplatelets, glucocorticoids, and SSRIs showed an increased risk for any bleeding and GI bleeding.

## Graphical abstract

Patients on nonvitamin K antagonist have an increase in the risk of bleeding when concomitantly exposed to antiplatelets, glucocorticosteroids, and selective serotonin reuptake inhibitors antidepressants.

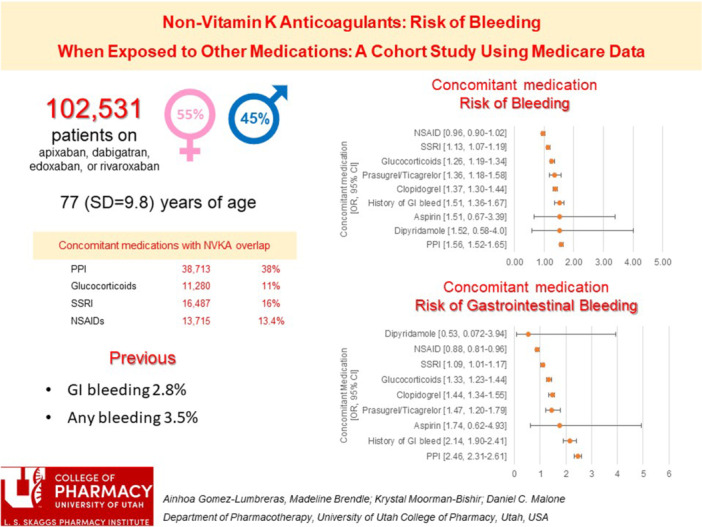

## 1 Introduction

After over 50 years of warfarin use for antithrombotic treatment and prophylaxis the first nonvitamin K antagonist (NVKA), dabigatran, was approved by the FDA in 2010 [1]. Since then, three other NVKAs have been approved—rivaroxaban, apixaban, and edoxaban. Compared to VKAs, NVKAs have shown to reduce the risk of bleeding while maintaining adequate antithrombotic efficacy in both clinical trials and real‐world studies [2, 3, 4]. In the last decade, NVKA use has increased worldwide due to their safety profile, with no monitoring and dose‐adjusting needed [5].

Polypharmacy is a common issue among elderly, with up to 44% prevalence, especially with those receiving NVKA [6, 7]. Previous research has reported rates of polypharmacy among these patients as high as 60% [8]. Polypharmacy is a risk factor for bleeding in the elderly due to potential drug–drug interactions [9, 10]. Patients with an indication for NVKAs may have concomitant exposure to antiplatelets (e.g., coronary heart disease), nonsteroidal anti‐inflammatory drugs (NSAIDs) (e.g., osteoarthritis, migraine, pain, etc.), serotonin selective reuptake inhibitors (SSRIs) (e.g., depression, anxiety, fibromyalgia… etc.), and proton pump inhibitors (PPIs) [11]. Drug interactions in anticoagulated patients are clinically relevant as these can increase either the risk of stroke (i.e., lack of efficacy) or the risk of bleeding [12].

Pharmacokinetic interactions lead to changes in plasma drug concentration, as the interaction can alter the NVKA metabolic pathway (i.e., CYP3A4/P‐glycoprotein) [13]. Antiepileptics, such as carbamazepine, induce CYP3A4 and are associated with lack of anticoagulant efficacy due to a decrease in anticoagulant levels [13, 14]. Pharmacodynamic interactions, due to an overlap in the mechanism of action, lead to an increased risk of bleeding [12]. Increased risk of bleeding has been shown for antiplatelets (e.g., clopidogrel, dipyridamole, prasugrel, etc.), NSAIDs (e.g., ibuprofen, diclofenac, indomethacin, etc.), SSRIs (e.g., paroxetine, fluoxetine, citalopram, etc.), and systemic glucocorticoids (e.g., dexamethasone, prednisone, etc.) [15, 16, 17, 18]. On the other hand, PPIs reduce the risk of gastrointestinal (GI) bleeding when used with oral anticoagulants [19, 20].

The purpose of this study was to assess the risk of bleeding among a Medicare population receiving NVKA and exposed to potentially interacting medications.

## 2 Methods

This study is a retrospective observational NVKA cohort study using Medicare data from 2017 to 2019. This manuscript follows the RECORD‐PE guideline for reporting pharmacoepidemiology studies using observational routinely collected health data [21]. To see the study flowchart design please see Supporting Information S1: Figure S1.

### 2.1 Source

This study was conducted using data from the Centers for Medicare and Medicaid Services (CMS) fee for service (FFS) claims. Data utilized in this study included the following: Medicare Beneficiary Summary File Base with Medicare Part A, B, C, and Medicare Part D Event (PDE). Variables of interest included chronic conditions, cost and healthcare utilization, inpatient and outpatient claims, and medication characteristics.

### 2.2 Inclusion/Exclusion Criteria

**Figure 1:**
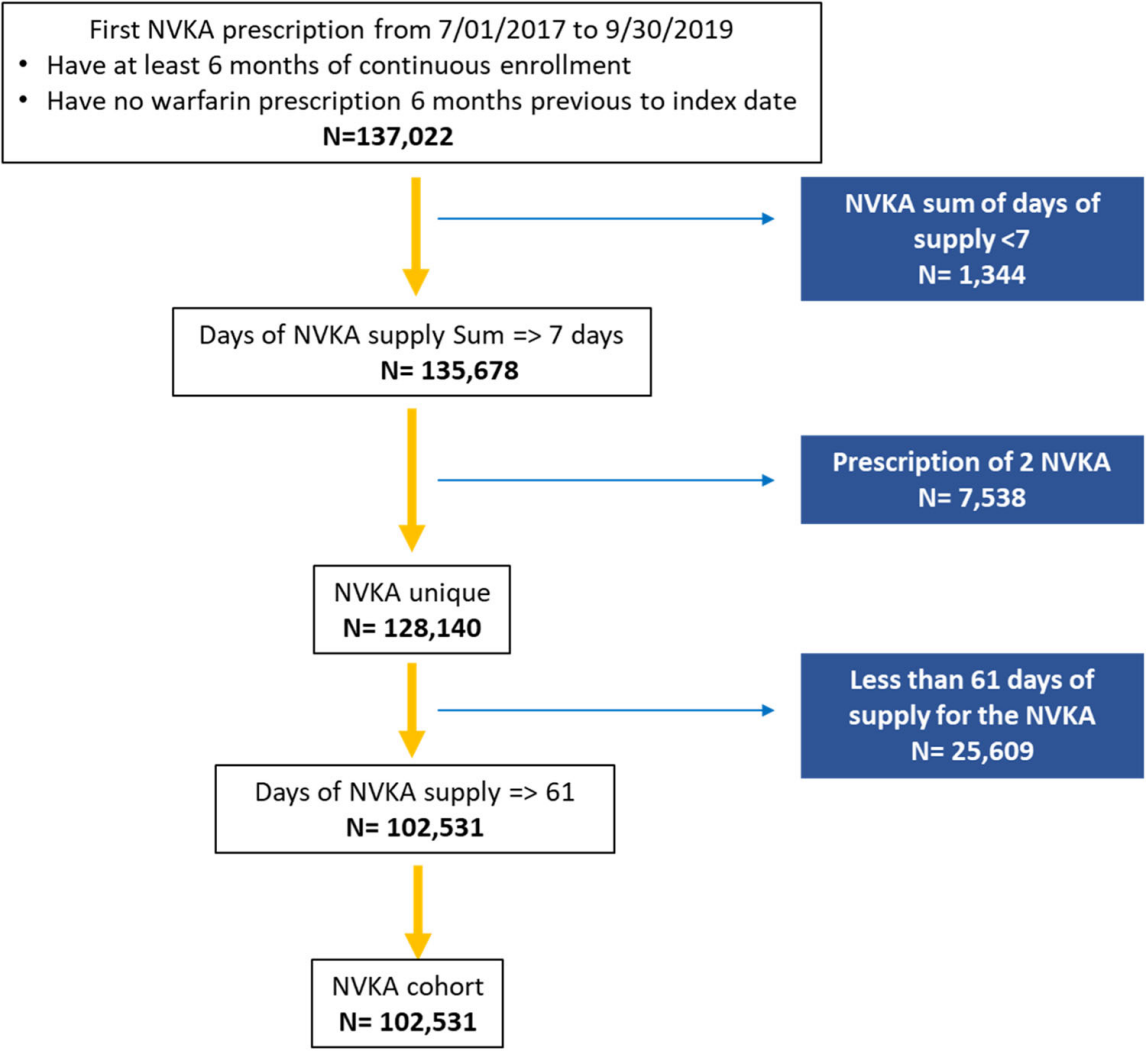
Flowchart cohort. NVKA, nonvitamin K antagonists.

Patients with at least one prescription for NVKAs (i.e., dabigatran, rivaroxaban, apixaban, or edoxaban), 6 months of continuous enrollment, and who were not utilizing warfarin within the 6 months before the index date were included. Patients with < 61 days of NVKA supply or who were receiving more than one type of NVKA were excluded. To see the cohort flowchart please see Figure 1.

### 2.3 Exposure

Cohort entry date was defined by the first NVKA prescription date until the last day of the supply. A concomitant overlapping period while on an NVKA was assessed for the following medications: aspirin, clopidogrel, dipyridamole, prasugrel/ticagrelor, systemic glucocorticoids, NSAIDs, PPIs, and SSRIs. See the Supporting Information S1: Table S1 for the specific medications for these four groups. First and last day of supply were used to assess concomitant exposure to any of these medications during the NVKA exposure.

### 2.4 Primary Outcome

The primary outcomes of interest were any bleeding event and a GI bleed after the index date. Bleeding events related to postprocedural or post‐surgery encounters were excluded. International Classification of Diseases, Ninth Revision, Clinical Modification (ICD‐9‐CM) and ICD‐10‐CM codes were used to identify bleeding events. See Supporting Information S1: Table S2 for the list of diagnosis codes used in this study.

### 2.5 Statistical Analysis

Descriptive statistics were used to describe the demographic and clinical characteristics of the cohort and NVKA treatment. Mean and standard deviations (SD) were reported for continuous variables including age and body mass index (BMI). Frequency and percentages were reported for categorical variables including sex, race, bleeding medical history, and concomitant medication use. Multivariable logistic regressions were used to determine the association between risk of any bleeding or GI bleeding and concomitant overlapping periods while on NVKA for the following medications: NSAIDS, SSRI, PPI, platelet aggregation inhibitors, and glucocorticoids. The logistic regression analysis was adjusted by race, gender, and age. Significance was considered if *p* < 0.05. Spearman's correlation coefficient was calculated to assess the relationship between PPI use and NSAID or SSRI use. All analyses were performed using SAS software version 9.4.

## 3 Results

The cohort included a total of 102 531 individuals receiving a NVKA. The cohort had a mean age of 77 years (SD 9.8), 55% were females (*N* = 56 671), and the majority were white (*N* = 90 128, 88%). Less than 5% of the cohort had history of bleeding (*N* = 3602, 3.5%) before initiating a NVKA. Polypharmacy was a common occurrence in this cohort with over a quarter concurrently receiving a PPI (*n* = 38 713, 38%), approximately 15% on SSRI and NSAIDS (*n* = 16 487, 16% and *n* = 13 715, 13.4%, respectively), but less than 1% on aspirin (*n* = 46, 0.04%). Complete cohort characteristics and concomitant medication exposures are shown in Table 1.

**Table 1:** Baseline characteristics for the nonvitamin K antagnonists cohort.

Total cohort (*N* = 102 531)	Mean or *N*	SD or %
Age (years)	77	9.8
Sex (Male)	45 860	45
Race		
White	90 128	88
Black	6788	7
Asian	1323	1.3
Hispanic	1393	1.4
North American Native	344	0.3
Other	1134	1.1
Unknown	1421	1.4
History of		
GI bleeding	2908	2.8
Any bleeding	3602	3.5
Concomitant medications with NVKA overlap		
Aspirin	46	0.04
Clopidogrel	15 795	15.4
Dipyridamole	31	0.03
PPI	38 713	38
Prasugrel/ticagrelor	1502	1.2
Glucocorticoids	11 280	11
SSRI	16 487	16
NSAIDs	13 715	13.4

Abbreviations: GI, gastrointestinal; N, number; NSAIDs, non‐steroidal anti‐inflammatory drugs; NVKA, nonvitamin K antagonist; PPI, proton pump inhibitors; SD, standard deviation; SSRI, selective serotonin reuptake inhibitors.

**Figure 2:**
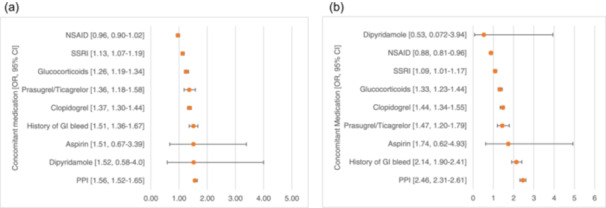
Risk of bleeding with concomitant exposure. (a) Risk of any bleeding for concomitant exposure. (b) Risk of GI bleeding for concomitant exposure. CI, confidence interval; GI, gastrointestinal; NSAIDS, nonsteroidal anti‐inflammatory drugs; OR, odds ratio; PPI, proton pump inhibitors; SSRI, selective serotonin reuptake inhibitors. Adjusted for race, sex, age and history of bleeding.

Risk for bleeding was increased for those subjects receiving clopidogrel (OR: 1.37, 95% CI: 1.30, 1.44) or prasugrel/ticagrelor (OR: 1.36, 95% CI: 1.18, 1.58) and glucocorticoids (1.26, 95% CI: 1.19, 1.34). SSRIs had an increased risk as well (OR: 1.13, 95% CI: 1.07, 1.19). Logistic regression‐adjusted results for any bleeding are shown in Figure 2a. GI bleeding was associated with concomitant exposure to NVKA and clopidogrel (OR: 1.44, 95% CI: 1.34, 1.55, prasugrel/ticagrelor (OR: 1.47, 95% CI: 1.20, 1.79), SSRIs (OR: 1.09, 95% CI: 1.01, 1.17) and glucocorticoids (OR: 1.33, 95% CI: 1.23, 1.44) (Figure 2b).

PPI use was correlated with both NSAID (*r* = 0.07, *p* ≤ 0.01) and SSRI use (*r* = 0.09, *p* ≤ 0.01). PPI concomitant use with NVKA was associated with any bleeding (OR: 1.56, 95% CI: 1.52, 1.65) or GI bleeding (OR: 2.46, 95% CI: 2.31, 2.61). This finding is likely confounding by indication, with persons at risk of bleeding being prescribed PPIs as a prophylactic measure to reduce the incidence of bleeding.

## 4 Discussion

This study documents that many individuals receiving NVKA are at risk of bleeding due to exposure to other medications that are well‐known to affect bleeding. Increase rates of any bleeding was observed among those receiving a NVKA and SSRIs, glucocorticoids, antiplatelets, and PPIs. When the outcome of interest was GI bleeding, concomitant use of NVKA and glucocorticoids, antiplatelets and PPIs were associated with an increased incidence of bleeding. It was not surprising to observe an increase in bleeding events because the cohort was an older Medicare population. Age is an independent risk factor for GI bleeding [22, 23]. The majority of patients on NVKA in the United States are older because the indications for NVKA are mostly age dependent, including nonvalvular atrial fibrillation and thromboembolic prophylaxis/treatment [24]. Age is also a risk factor for polypharmacy and increases the propensity for drug–drug interactions [25, 26]. Physiologic changes that are usually not accounted for in the elderly may also contribute to an increase of adverse events due to medications [27, 28].

The mechanism of action for the pharmacodynamic interactions observed in this study are briefly presented here. It is generally obvious that the use of antiplatelets products among patients on an oral anticoagulant will increase the risk of bleeding. This has been studied in numerous trials [29, 30]. Except for dipyridamole, we found risk for any bleeding and GI bleeding with concomitant use of antiplatelets medications. Clopidogrel is a second‐generation P2Y12 inhibitor and is more commonly used than prasugrel or ticagrelor (third‐generation ones) [31]. Clinical trials have shown higher risk of bleeding for the third‐generation P2Y_12_ inhibitors [32, 33]. Observational studies comparing clopidogrel versus prasugrel/ticagrelor showed fewer GI bleeding events with prasugrel/ticagrelor. However, when accounting for major bleeding hospitalization, prasugrel showed higher risk of bleeding than clopidogrel [34, 35]. It is important to note that this study could not evaluate the role of aspirin, another antiplatelet medication, because it is available over‐the‐counter and not in the Medicare Part D claims.

With respect to SSRIs, the mechanism of action is likely due to the inhibition of serotonin needed for platelet activation [36, 37]. SSRIs are commonly use in the elderly [38]. The risk of bleeding when concomitantly taking an SSRI and anticoagulant has been shown in a systematic review and meta‐analysis (any bleeding: OR: 1.39, 95% CI: 1.24, 1.55; GI bleeding: OR: 1.34, 95% CI: 1.19, 1.59) [39].

Glucocorticoids alone have previously shown risk for GI bleeding; however, the role of glucocorticoids increasing risk of bleeding is controversial [40, 41, 42]. In our cohort, risk for any bleeding and GI bleeding was shown with concomitant exposure to glucocorticoids. A meta‐analysis found risk of GI bleeding when on corticosteroids excluding previous peptic ulcer disease or concomitant exposure to aspirin or NSAIDs [17]. When glucocorticoids are used concomitantly with an SSRI, there is a significant increase in the risk of upper GI bleeding (HR: 3.454, 95% CI: 2.9, 4.4) [43]. Our study found an increased risk of bleeding for patients on NVKA with exposure to glucocorticoids.

Surprisingly our study did not find an increased risk of bleeding with concomitant exposure to NSAIDs. Concomitant use of NSADs and prednisone with NVKA have been described up to rates of 50% and an increase on the risk of bleeding [44, 45]. Another consideration is that NSAIDs can be acquired as over‐the‐counter medications [46]. A case‐crossover study conducted in the French nationwide health database found risk of GI bleeding when concomitant use of NSAIDs and anticoagulants (OR: 3.59, 95% CI: 1.58, 8.17) and also risk of non‐GI bleeding (OR: 2.72, 95% CI: 1.23, 6.04) [47].

The medication category with the highest concomitant exposure in the cohort was PPIs (38%, *N* = 38 713). Previous studies of NVKA users have found that up to 46% have also been receiving a PPI [48, 49]. PPIs reduce the risk of GI bleeding, but over use of PPIs among population in general has prompted the development of guidelines to reduce use of PPIs [19, 20, 49, 50]. In our analysis, PPI use was associated with an increased risk of bleeding. As mentioned above, this increased risk can be explained by a confounding bias, as use of PPIs is more frequent among higher risk patients, an issue that has been mentioned in other observational studies [49].

### 4.1 Limitations

Our research has the inherent limitations previously described for pharmacoepidemiology studies using secondary health databases, such as missing data, recording errors, and exposure [51]. We did not have data on the specific strength/dose and duration on the assessed drugs, and did not attempt to assess time to event. The potential indication bias for the concomitant exposure of PPI couldn't be addressed as we did not have information to adjust our results, that may explain the risk of bleeding and GI bleeding observed for concomitant use of PPIs, however, we tried to adjust using one of the more relevant risk factors, previous bleeding [52]. Other potential risk factors for bleeding such as clinical attributes like renal function, blood pressure, liver function, were not available in our data and therefore were not included. Another limitation is the generalizability of the findings to other countries should be done with caution. This was a real‐world study with a large sample size of patients from the Medicare database which is representative of an older population of the United States.

## 5 Conclusion

NVKA inherent risk of bleeding makes them more prone to drug interactions with a potential increase of this risk. Patients on NVKA are at increased risk for any bleeding (GI bleeding included) when taking antiplatelets, glucocorticoids, and SSRIs. Use of PPIs may be a confounding medication because it does not increase risk of bleeding but is associated with the use of increased bleeding risk medications. Specific research accounting for patient‐specific criteria, amount of concomitant exposure and time to event are needed to a more specific and establish risk of bleeding.

## Author Contributions

Ainhoa Gomez‐Lumbreras conceived and designed the analysis and drafted the paper. Madeline Brendle performed the analysis and reviewed and contributed to the draft. Krystal Moorman‐Bishir conceived and designed the analysis, and reviewed and contributed to the draft. Malinda Tan conceived and designed the analysis, performed the analysis and reviewed and contributed to the draft, Daniel C. Malone conceived and designed the contributed analysis tools, and reviewed and contributed to the draft. He supervised the research project and manuscript preparation.

## Ethics Statement

The University of Utah IRB acknowledges the conduction of this research and exempt from informed consent (IRB_00135873 Evaluation of Medication Safety and Adverse Events in Medicare Population).

## Conflicts of Interest

The authors declare no conflicts of interest.

## Supporting information

Supporting information.

## Data Availability

The data that support the findings of this study are available from the corresponding author upon reasonable request.
