# Supplementary material for: Nonvitamin K Anticoagulants: Risk of Bleeding When Interacting With Other Medications: A Cohort Study From Medicare
Source: Clin Cardiol. 2024 Oct 3;47(10):e70023. doi: 10.1002/clc.70023 (PMC11447635; doi:10.1002/clc.70023)
Supplement: Supplementary file 1 — Supporting information. [file CLC-47-e70023-s001.docx]

Figure 1S Flowchart design


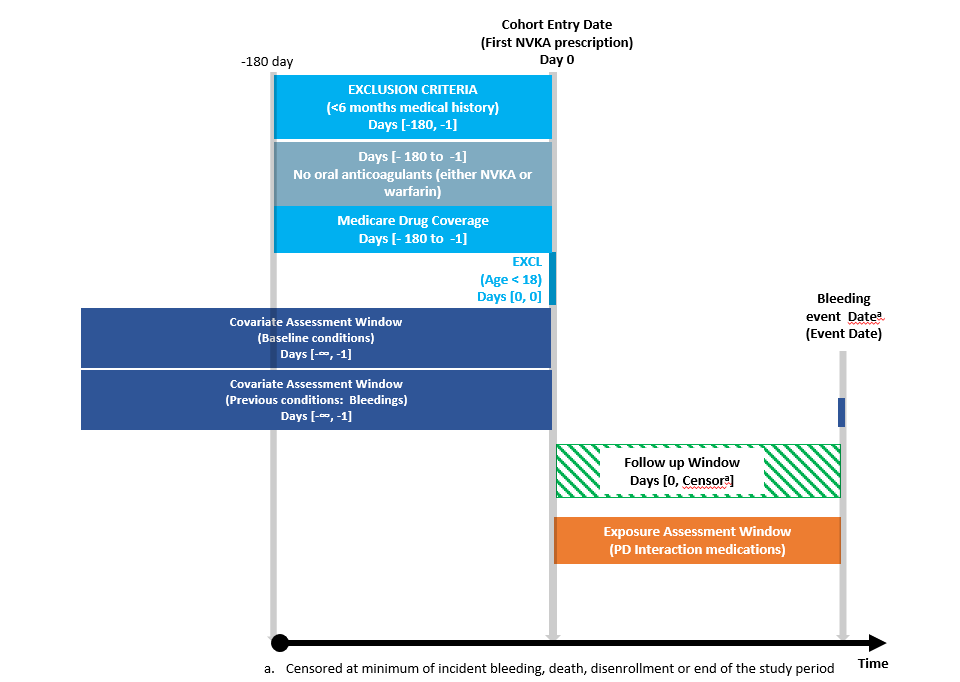


NVKA Non Vitamin K Antagonist; PD pharmacodynamic;

Table S1.

List of specific agents of the following groups: systemic glucocorticosteroids, NSAIDs, PPIs and SSRI groups of interest

| **Group** | **Drug** |
| --- | --- |
| Systemic glucocorticoids | Deflazacort  Dexamethasone  Fludrocortisone  Fluocinonide  Methylprednisolone  Oxandrolone  Prednisolone |
| NSAID | Celecoxib  Diclofenac  Ibuprofen  Indomethacin  Ketoprofen  Ketorolac  Meloxicam  Naproxen  Piroxicam |
| PPI | Omeprazole  Pantoprazole  Lansoprazole  Rabeprazole  Esomeprazole |
| SSRI | Citalopram  Escitalopram  Fluoxetine  Fluvoxamine  Paroxetine  Sertraline |

NSAID: Non-steroidal anti-inflammatory drugs; SSRI: selective serotonin reuptake inhibitors;

Table S2. ICD 9-10^th^ codes for bleeding events

| **Any bleeding codes** | **Gastrointestinal bleeding** |
| --- | --- |
| 'K921','K922','K5731','K625','K31811','K920','K661','K2971','K2211','K5521','K264','K254','K2981','K226','K6381','K3182','K2901','K274','K5711','K5793','K51911','K5791','K282','K276','K272','K266','K262','K256','K252','K250','K5781','K260','K270','K5751','K280','K5741','K284','I8511','K5733','K5721','K5713','K51811','K51411','K51211','K50911','K51011','K51311','K50811','K51511','K50111','K5701','I8501','K2991','K5753','K2961','K2951','K2941','K2931','K2921','K286','R040','R319','R042','R310','N939','R58','N938','R233','N950','R0489','I312','M25012','M25062','H4311','H05232','M25073','M25071','M25069','M25061','M25059','M25051','M25042','M25039','M25032','M25031','M25022','M25019','M25011','M2500','H05233','M25021','M25029','H4312','M25041','M25049','M25052','M25072','M25074','H4310','R049','R041','N99530','N99520','N924','N421','M2508','H05231','M25076','H05239','H4313','M25075','S065X0A','I615','S066X0A','I6201','I618','I610','I609','S065X9A','I629','I619','I614','I611','S066X9A','I6200','I6202','I6203','I608','S06350A','S065X7A','S066X1A','I613','S065X1A','S06360A','S064X0A','S065X8A','S066X8A','S06358A','S064X9A','S066X3A','I6011','I606','S066X7A','S066X4A','S066X2A','I6052','S066X5A','I6002','I6051','S065X6A','S065X5A','I6010','I607','S065X3A','S065X2A','I6012','S064X7A','S064X5A','S064X3A','I604','S064X1A','S06369A','S06367A','S06366A','S06365A','S06363A','S06362A','S06361A','S06359A','S06357A','S06355A','I6000','I6031','S06354A','S06353A','S06351A','S06349A','S06352A','S06348A','S06356A','S06347A','I6030','S06346A','S06345A','I6032','S06364A','I621','S06368A','S06344A','S06343A','S06342A','S06341A','I6001','S064X2A','S064X4A','S064X6A','I6050','S064X8A','I6022','I616','S06340A','S065X4A','I6021','S066X6A','I6020','I612' | 'K921','K922','K5731','K625','K31811','K920','K661','K2971','K2211','K5521','K264','K254','K2981','K226','K6381','K3182','K2901','K274','K5711','K5793','K51911','K5791','K282','K276','K272','K266','K262','K256','K252','K250','K5781','K260','K270','K5751','K280','K5741','K284','I8511','K5733','K5721','K5713','K51811','K51411','K51211','K50911','K51011','K51311','K50811','K51511','K50111','K5701','I8501','K2991','K5753','K2961','K2951','K2941','K2931','K2921','K286' |
